# Cultivation-independent methods applied to the microbial prospection of oil and gas in soil from a sedimentary basin in Brazil

**DOI:** 10.1186/2191-0855-1-35

**Published:** 2011-10-22

**Authors:** Paula B Miqueletto, Fernando D Andreote, Armando CF Dias, Justo C Ferreira, Eugênio V dos Santos Neto, Valéria M de Oliveira

**Affiliations:** 1 Division of Microbial Resources, Research Center for Chemistry, Biology and Agriculture (CPQBA), UNICAMP, CP 6171, CEP 13081-970, Campinas, SP, Brazil; 2 Departament of Soil Sciences, ESALQ, University of São Paulo, CP 09, CEP: 13418-900, Piracicaba, SP, Brazil; 3 PETROBRAS R&D Center, Cidade Universitária, Ilha do Fundão, Rio de Janeiro, RJ, CEP 21949-900, Brazil; 4 Biomedical Sciences Institute (ICB-IV), University of São Paulo, São Paulo, Brazil

**Keywords:** Short-chain hydrocarbons, Microbial prospection, Community structure, Gene libraries, Soluble di-iron monooxygenases

## Abstract

The upper parts of oil field structures may leak gas which is supposed to be indirectly detected by the soil bacterial populations. Such microorganisms are capable of consuming this gas, supporting the Microbial Prospection of Oil and Gas (MPOG) methodology. The goal of the present work was to characterize microbial communities involved in short-chain alkane metabolism, namely methane, ethane and propane, in samples from a petroliferous (P) soil through clone libraries of the 16S rRNA gene of the Domains Bacteria and Archaea and the catabolic gene coding for the soluble di-iron monooxygenase (SDIMO) enzyme alpha subunit. The microbial community presented high abundance of the bacterial phylum Actinobacteria, which represented 53% of total clones, and the Crenarchaeota group I.1b from the Archaea Domain. The analysis of the catabolic genes revealed the occurrence of seven Operational Protein Families (OPF) and higher richness (Chao = 7; Ace = 7.5) and diversity (Shannon = 1.09) in P soil when compared with a non-petroliferous (Np) soil (Chao = 2; Ace = 0, Shannon = 0.44). Clones related to the ethene monooxygenase (EtnC) and methane monooxygenase (MmoX) coding genes occurred only in P soil, which also presented higher levels of methane and lower levels of ethane and propane, revealed by short-chain hydrocarbon measures. Real-time PCR results suggested that the SDIMO genes occur in very low abundance in the soil samples under study. Further investigations on SDIMOs genes in natural environments are necessary to unravel their still uncharted diversity and to provide reliable tools for the prospection of degrading populations.

## Introduction

Surface geochemical petroleum exploration is defined as the search for migrated surface hydrocarbons and their alteration products, including changes in vegetation and microbial populations [Hitzman et al. 2009; Rashed et al. 2008; Davis and Updegraff 1954; Schumacher 2000]. Microbes play a profound role on the oxidation of migrating hydrocarbons, and are directly responsible for many surface manifestations of petroleum seepage. In this context, the Microbial Prospection for Oil and Gas (MPOG), developed in Germany and used as a stand-alone technique for detecting microseepages since 1961, is based on the knowledge that oil and gas fields emit a continuous stream of light-hydrocarbon gases towards the Earth's surface [Schumacher 2000; Wagner et al. 2002; Tucker and Hitzman 1996].

Specialized microorganisms, such as the hydrocarbon-oxidizing bacteria, depend on light-hydrocarbon gases as their only energy source [Wagner et al. 2002]. In terms of investigation aiming at the microbial hydrocarbon prospection, two groups are relevant: gram-positive bacteria, represented mainly by Actinobacteria from CRNM complex (*Corynebacterium, Rhodococcus, Nocardia *and *Mycobacterium*) that uses short-chain hydrocarbons (C2-C8) as an energy source, and gram-negative bacteria, mainly the genus *Pseudomonas *that possesses the ALK system responsible for alkane degradation [Ginkel et al 1987; Shennan 2006; Kotani et al. 2006].

The Microbial Oil Survey Technique (MOST), developed by Phillips Petroleum Company, is one of the exploration methods and has been available to the petroleum industry since 1985 [Hitzman et al. 2009]. This methodology is based on the isolation of microorganisms on agar plates containing selective growth medium and subsequent counting of colony-forming units. Microbial anomalies have been proven to be reliable indicatives of oil and thermogenic gas occurrences in the subsurface, and the method has been widely used throughout the world. Generally, these methods involve microbial activity analysis in samples taken from depths of 0.2 to one meter [Shennan 2006].

Light hydrocarbon gases that migrate upward from buried reservoirs and become adsorbed to near-surface soils and sediments also represent a useful tool for oil and gas prospection, constituting the Sorbed Soil Gas (SSG) technique [Hitzman et al. 2009]. Areas of microseepage are detected by observing the concentration and composition of light hydrocarbons, chiefly methane through butane, extracted from these soils and sediments. These exploration methods can be used in combination with other data, such as geological and geophysical, to reduce exploration costs and increase success rates [Schumacher 2000]. According to Hitzman (2009), prospections associated with microseepage anomalies are 4-6 times more likely to result in a commercial discovery than prospects with no associated seepage anomaly.

In spite of the well established exploration methods, it is important to emphasize that all of the techniques applied for such goals have been, for many years, based on traditional microbiological methods, encompassing microbial isolation and colony-forming units counting.

Provided the knowledge that only a very small fraction of microorganisms can be recovered in laboratory, this work aimed at a cultivation-independent characterization of the microbial community from a petroliferous basin area explored by PETROBRAS (Brazil), as well as from a randomly selected non-petroliferous area. We also focused on specialized populations of microorganisms, the short chain hydrocarbon-oxidizing bacteria, which are directly involved in light gas metabolism. Soluble di-iron monooxygenases (SDIMOs) were the group of enzymes chosen as a target in order to detect these specialized populations. SDIMOs are represented by multicomponent enzymes that catalyze the initial oxidation of hydrocarbons in phylogenetically and physiologically diverse bacteria [Coleman et al. 2006]. Their structure include a minimum of four proteins; a hydroxylase with two or three subunits, an effector (or coupling) protein and a reductase [Leahy et al. 2003]. The presently biochemically characterized SDIMOs belong to six distinct lineages. The enzymes of subgroups 1 and 2 function predominantly as aromatic monooxygenases and the ones of groups 3-6 as aliphatic monooxygenases. The SDIMOs have numerous applications in bioremediation and biocatalysis [Holmes and Coleman 2008].

## Materials and methods

### Sampling and area background

Soil samples were provided by CENPES/Petrobras (Rio de Janeiro, Brazil). Sampling was performed in September of 2007 on the terrestrial petroliferous field *Jaçanan*, located in Potiguar Basin (Brazil), with the use of appropriate sampling equipment at a 70 cm depth and following strict procedures to avoid contaminations. Samples were then immediately transferred to metal containers and hermetically closed for subsequent gas analysis from the headspace compartment. Sample from this area was named "Petroliferous Soil (P Soil)". Non-petroliferous soil was collected in March of 2007 in the surroundings of Lorena city (Southeast, Brazil) and the same methodology was used to collect and store the samples.

The *Potiguar *sedimentary basin, located in northeast Brazil, is an area of growing exploration interest. Exploration in the basin began onshore in 1956 and nowadays it has approximately 70 oilfields, 58 of which are onshore and account for 85% of the basin's production. The basin currently produces approximately 125,000 barrels of oil equivalent per day, making it the second most important producing area in Brazil after the Campos basin [Lovatini et al. 2010].

### Gas Chromatography Analysis

Gas analysis of the soil samples was carried out at PETROBRAS R&D Center (Rio de Janeiro, Brazil). The quantitative analysis of hydrocarbons in the C_1 _to C_5 _interval were performed by a Hewlett-Packard 5890 series II gas chromatograph equipped with a flame ionization detector (FID) heated at 250°C. A fused silica HP-5-MS capillary column (50 m length, 320 μm ID, 8 μm film thickness) was used. The oven temperature was programmed from 50°C to 200°C with an initial isotherm of 1.75 min and a heating rate of 15°C/min. Helium was used as carrier gas at a constant flow of 8.8 mL/min and quantification was performed using an external calibration with standard gas mixture.

### DNA Extraction

DNA extraction from soil samples was carried out using the PowerSoil™ DNA Isolation Kit (Mo Bio Laboratories), according to the manufacturer's instructions. The yield and integrity of the environmental DNA obtained were confirmed through electrophoresis in 1% agarose gel.

### 16S rRNA Gene Libraries

Two 16S rRNA gene libraries were constructed from the sedimentary soil sample (P soil), one for each domain, Bacteria and Archaea. For the bacterial 16S rRNA gene library construction, the amplification was performed using the primer set 10f and 1100r [Lane 1991]. Fifty μl (50 μl) reaction mixtures contained 50-100 ηg of total DNA, 2 U of *Taq *DNA polymerase (Invitrogen), 1× *Taq *buffer, 1.5 mM MgCl_2_, 0.2 mM of dNTP mix (GE Healthcare) and 0.4 mM each primer. The PCR amplifications were done using an initial denaturation step of 2 min at 95°C, followed by 30 cycles of 1 min at 94°C, 1 min at 55°C, and 3 min at 72°C, followed by a final extension step at 72°C for 3 min, in an Eppendorf thermal cycler. The archaeal 16S rRNA amplification was performed using the primer set ARCH344f [Casamayor et al. 2002] and ARCH1400r [Kudo et al. 1997] and the following amplification program: an initial denaturation of 94°C for 1.5 min; nine cycles of 30 s at 94°C, 30 s at 67°C (decreasing 0.5°C each cycle) and 1.5 min at 72°C for extension; followed by other 25 cycles of denaturation at 94°C for 30 s, 62°C for 30 s, 72°C for 1.5 min and a final extension step of 10 min at 72°C.

### SDIMO Gene Libraries

A nested PCR strategy and degenerate primers were employed for the amplification of the gene coding for the alpha subunit of soluble di-iron monooxygenase enzymes from the environmental samples and subsequent library construction. In this case, one gene library was assembled for each soil sample, the petroliferous (P soil) and non-petroliferous soil (Np soil). In the first PCR reaction total genomic DNA from soil sample was used as template and the primer set NVC58 and NVC65 [Coleman et al. 2006] was used, yielding a fragment with an expected of 1,100 bp. The PCR product from the first reaction was then employed as template for the second PCR amplification using the primer set NVC66 and NVC57 [Coleman et al. 2006], which yielded a smaller fragment with an expected size of approximately 410 bp. The primers were designed to target conserved regions of the gene coding for the alpha subunit of subgroups 3, 4, 5 and 6, which are involved in the degradation of alkenes and propane. PCR conditions were as described in Coleman et al. (2006).

### Cloning and Sequencing of PCR Products

PCR replicates of each target gene were pooled, purified using GFX PCR DNA and Gel Band Purification kit (GE Healthcare), according to the manufacturer's protocol, and concentrated in a speed vacuum concentrator 5301 Eppendorf, A-2-VC rotor. The purified PCR product (200 ηg) was cloned into a pGEM-T Easy Vector (Promega), according to the manufacturer's instructions, and transformed into *E. coli *JM109 competent cells. Approximately 200 positive clones were selected from each library for subsequent sequencing. The 16S rDNA and SDIMO inserts were amplified from plasmid DNA of selected clones using the universal the M13 forward (5'-CGCCAG GGT TTT CCC AGT CAC GAC-3') and reverse (5'-TTT CAC ACA GGA AAC AGC TAT GAC-3') primers. PCR was performed in a 50-μl reaction volume, containing 1-2 μl of an overnight clone culture, 0.4 μM of each primer, 0.2 mM dNTP mix, 2 U *Taq *DNA polymerase (Invitrogen), 1× *Taq *buffer, and 1.5 mM MgCl_2_. The amplification program consisted of an initial denaturation step at 94°C for 3 min, followed by 30 cycles of 94°C/20 s, 60°C/20 s, and 72°C/90 s. PCR products were purified as previously described for automated sequencing in the MegaBace DNA Analysis System 500 (GE Healthcare). The sequencing was carried out using 10f and 1100r primers for bacterial rRNA 16S; 344f and 1400r for Archaeal rRNA 16S, and NVC57 and NVC66 for the catabolic genes.

### Phylogenetic Analysis

Partial 16S rRNA sequences obtained from clones were assembled in a contig using the phred/Phrap/CONSED program [Ewing et al. 1998]. Identification was achieved by comparing the contiguous 16S rRNA sequences obtained with 16S rRNA sequence data from reference type strains, as well as environmental clones available in the public databases GenBank (http://www.ncbi.nlm.nih.gov) and RDP (Ribosomal Database Project - Release 10; http://rdp.cme.msu.edu/) using the BLASTn and Classifier routines, respectively. The sequences were aligned using the CLUSTAL X program [Thompson et al. 1997] and analyzed with MEGA software v.4 [Kumar et al. 2001]. Evolutionary distances were derived from sequence-pair dissimilarities calculated as implemented in MEGA, using Kimura's DNA substitution model [Kimura 1980]. For the identification of catabolic gene sequences BLASTx routine was used and nucleotide sequences coding for the alpha subunit SDIMO gene were translated *in silico*. The phylogenetic reconstruction was done using the neighbor-joining (NJ) algorithm, with bootstrap values calculated from 1,000 replicate runs, using the routines included in the MEGA4 software.

### Statistical Analysis

Diversity index calculations (α-diversity measures) were performed individually for all gene libraries with the programs MOTHUR version 1.13.0 and DOTUR v. 1.3 [Schloss & Handelsman 2005]. DOTUR was used to assign bacterial and archaeal 16S rRNA sequences to OTUs and to calculate rarefaction curves using 4 distance levels among sequences (3, 5, 10 and 20%), and to calculate, for each community, the Shannon (H') diversity index, Simpson index [Magurran 2004], and the nonparametric richness estimators ACE (ctimator) [Chao 1993] and Chao1 [Chao 1984]. Chao1 richness estimates were based on singletons and doubletons as described by Chao (1984), and ACE was based on the distribution of abundant (>10) and rare (≤10) species. MOTHUR was used for the analysis of SDIMO gene sequences, supporting the definition of Operational Protein Families (OPF) at a distance level of 83%.

### Quantitative PCR

Real time PCR was carried out in an Applied Biosystems thermo cycler (Step One Real Time PCR System). The reaction mixture was prepared using the Superscript^® ^III Platinum^® ^SYBR^®^Green One-Step qPCR Kit w/ROX (Invitrogen), according to the manufacturer's recommendations. The primer set BacqPCR - P1 (5' -CCT ACG GGA GGC AGC AG - 3') and BacqPCR - P2 (5' - ATT ACC GCG GCT GCT GG - 3') was employed for the bacterial 16S rDNA amplification, and the primer set NVC57 and NVC66 [Coleman et al. 2006] was used for the catabolic gene amplification. The real-time PCR program for the target DNA amplification was performed as follows: 95°C for 2 min followed by 30 cycles of 1 min at 94°C, 1 min at 55°C and 3 min at 72°C for extension. Bio-Rad iQ5 Optical System software (version 1.0, 2005) was used to analyze the amplification data.

### Standard Curve of real-time PCR

PCR products containing SDIMO and 16S rRNA gene fragments were used separately for each standard curve construction. Tenfold serial dilution (from 10^-1 ^to 10^-7^) was used in triplicate as PCR templates in order to produce curves to enable the quantification of total populations of bacteria as well as of short-chain hydrocarbon degrading microorganisms.

### Nucleotide sequence accession numbers

The 16S rRNA and SDIMO gene partial sequences determined in this study for the environmental clones were deposited at the Genbank database under the accession numbers JF681794 to JF681937 and JF694327 to JF694383 for the 16S rRNA from bacteria and archaea respectively and the numbers JN116289 to JN116426 for the SDIMO gene.

## Results

### Chromatographic analysis

Chromatographic data revealed relatively high concentration of methane in the soil sample from the petroliferous field (P soil) in comparison with ethane and propane contents (Table 1). For a comparison purpose, one soil sample from a different area, named "Non-petroliferous soil (Np Soil)", was submitted to gas chromatography analysis as well. This sample was collected in a region outside the sedimentary basin and represents a type of soil that would, in thesis, harbor lower levels of short-chain hydrocarbons, especially those related to the presence of petroleum at the subsurface of the studied area. These qualitative data function as an information input for our analysis, since it gives an idea of light gas levels that one can expect to find in samples with different characteristics such as these. The present analysis showed that, surprisingly, the sample representing the non-petroliferous area contained higher concentrations of ethane and propane, components of natural gas from petroleum, in comparison with those found in the P soil (Table 1).

**Table 1:** Measures of short-chain hydrocarbons contents taken from the headspace of Np Soil and P Soil containers (ppm).

	P Soil	Np Soil
Methane	1152.14	140.36
Ethane	1.16	5.36
Propane	0.39	3.43

### Composition of the soil communities

**Figure 1:**
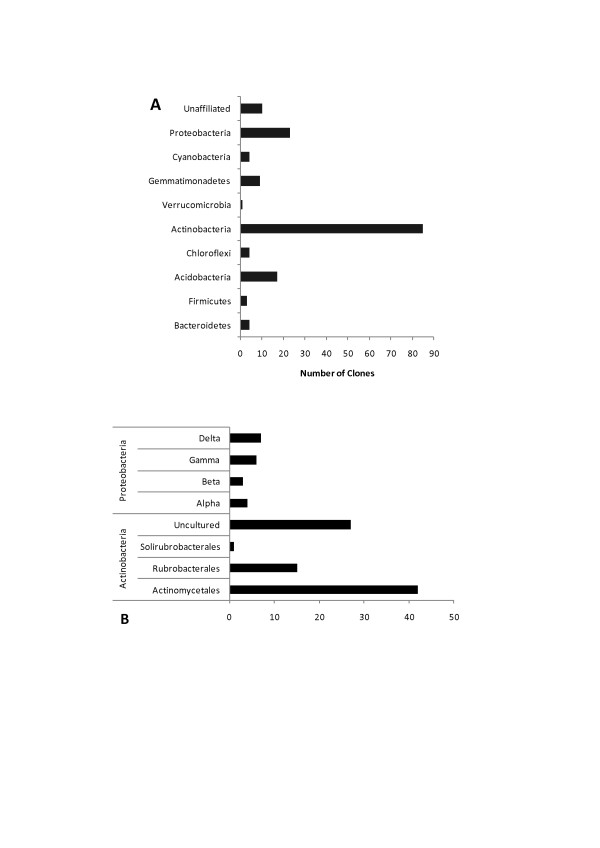
**Proportion of 16S rRNA gene clones from P Soil among bacterial phyla (A); among classes within the phylum Proteobacteria and among orders within the phylum Actinobacteria (B)**. Sequence classification was based on the RDP classifier and BLASTn results.

The composition of bacterial and archaeal communities was determined by analysis of 16S rRNA gene clone libraries. A total of 160 partial sequences were obtained from the 16S rRNA bacterial library and 60 from the archaeal library, each consisting of 850 nucleotides in average. These 16S rRNA sequences were compared with sequences from reference and type strains, as well as environmental clones, available at the GenBank and RDP II databases. Ten major bacterial taxa were identified (Figures 1A and 1B), being the phylum Actinobacteria the most abundant one, accounting for 52.2% of all bacterial clones distributed among the orders Actinomycetales (25%), Rubrobacterales (9.5%) and Solirubrobacterales (0.7%). Clones classified as uncultured Actinobacterium accounted for 17% of the bacterial library (Figure 1A).The second most abundant was the phylum Proteobacteria, which represented 12.5% of the bacterial library (2.5% affiliated to Alphaprotebacteria, 2% to Betaproteobacteria, 3.7% to Gammaproteobacteria and 4.3% to Deltaproteobacteria); followed by Acidobacteria representing 11% of total clones. The remaining sequences were significantly less numerous, being related to the phyla Gemmatimonadetes (5.8%), Bacteroidetes (2.5%), Chloroflexi (2.5%), Cyanobacteria (2.5%), Firmicutes (2%) and Verrucomicrobia (0.6%). Some sequences (6.4%) could not be affiliated to any known taxa (Figures 1A and 1B).

**Figure 2:**
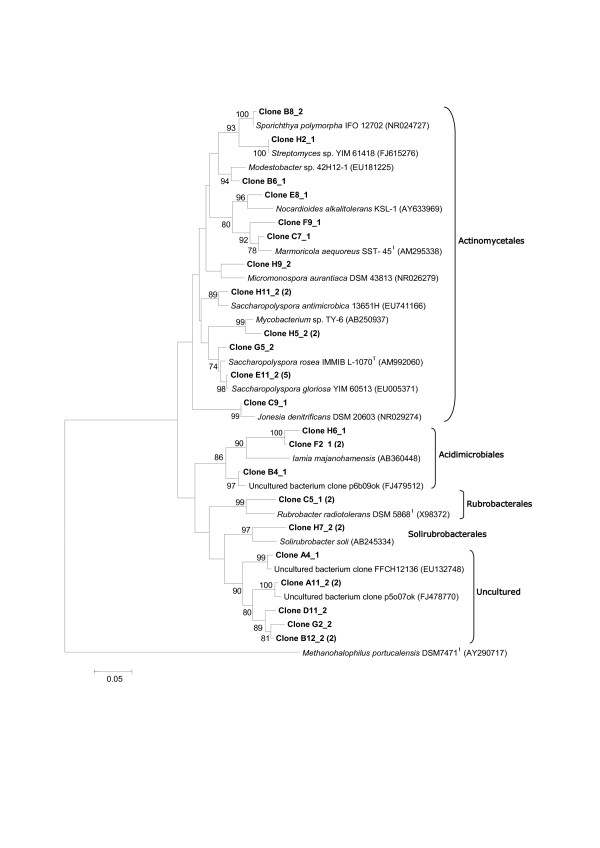
**Phylogenetic analysis based on partial 16S rRNA sequences from the P Soil clone library representing members of phylum Actinobacteria and related species**. Evolutionary distances were based on the Kimura 2p model and tree reconstruction on the neighbor-joining method. Bootstrap values (1,000 replicate runs, shown as %) greater than 70% are listed. GenBank accession numbers are listed after species names. *Methanohalophilus portucalensis *was used as the outgroup.

**Figure 3:**
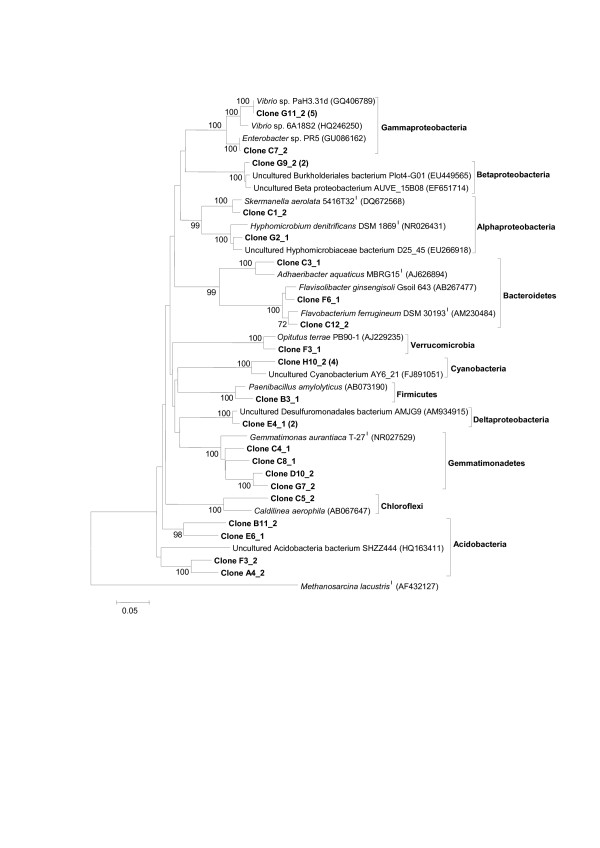
**Phylogenetic analysis based on partial 16S rRNA sequences from P Soil clone library representing members of major phyla in the Bacteria Domain, except Actinobacteria, and related species**. Evolutionary distances were based on the Kimura 2p model and tree reconstruction on the neighbor-joining method. Bootstrap values (1,000 replicate runs, shown as %) greater than 70% are listed. GenBank accession numbers are listed after species names. *Methanosarcina lacustris *was used as the outgroup.

Phylogenetic analysis allowed identification of many bacterial members of the soil sample at the species level (Figures 2 and 3). The majority of Actinobacteria-related clones occurring in the 16S rDNA library from the petroliferous soil were clearly related to the order Actinomycetales (Figure 2), which includes species from the CNMR complex, such as *Mycobacterium *sp. TY-6. Several clones were closely related to species like *Sporichthya polymorpha, Saccharopolyspora antimicrobica, Saccharopolyspora gloriosa*, *Nocardioides alkalitolerans *and *Marmoricola aequoreus*. Rubrobacterales was the second more abundant order within the phylum Actinobacteria, represented in the tree by three clones closely related to *Rubrobacter radiotolerans*. The orders Solirubrobacterales and Acidimicrobiales were also identified in the sample and were represented by the species *Solirubrobacter soli *and *Iamia majanohamensis*, respectively.

Clones affiliated to Proteobacteria were closely related to representatives of each class of the group, supported by high bootstrap values (100%), as shown in Figure 3. Deltaproteobacteria was represented by the order Desulfuromonadales. Several 16S rDNA sequences were affiliated to Gammaproteobacteria, identified as *Vibrio *sp., while sequences affiliated to Betaproteobacteria were related mainly to the order Burkholderiales. Some clones affiliated to the class Alphaproteobacteria were identified at the species level, such as *Hyphomicrobium denitrificans *and *Skermanella aerolata*.

Other bacterial species identified in this study included *Paenibacillus amylolyticus*, affiliated to Firmicutes, *Caldilinea aerophila*, representing the Chloroflexi phylum, and *Opitutus terrae*, belonging to the phylum Verrucomicrobia. The phylum Bacteroidetes was represented by clones related to the species *Flavisolibacter ginsengisoli*, *Flavobacterium ferrugineum*, and *Adhaeribacter aquaticus.*

The phylum Gemmatimonadetes was represented in the P Soil 16S rDNA library by four clones identified as *Gemmatimonas aurantiacus.*

**Figure 4:**
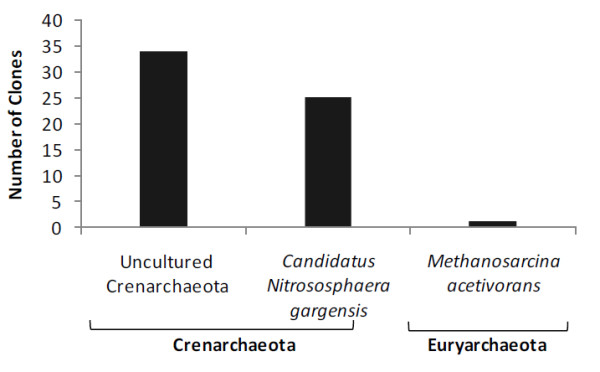
**Proportion of 16S rRNA gene clones from P Soil among archaeal phyla**. Sequence classification was based on the RDP classifier and BLASTn results.

The sequencing of the 16S rRNA archaeal library revealed that 56% of the community in P soil was represented by the Crenarchaeota phylum, with clones being identified as uncultured Crenarchaeota (Figure 4). Approximately 42% of total clones were included in the category *Candidatus nitrososphaera gargensis*, currently classified as a member of the phylum Thaumarchaeota. Only 2% of the sequences were afilliated to a species from the Euryarchaeota group, *Methanosarcina acetivorans*, a methanogenic organism.

### SDIMO gene diversity in soil samples

**Figure 5:**
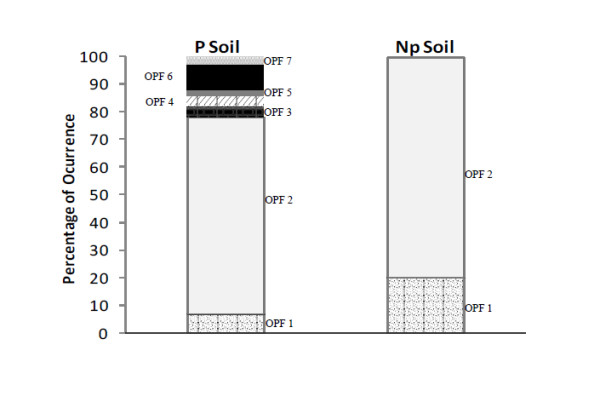
**Soluble monooxygenase alpha subunit gene frequency and OPF identification in soil samples from petroliferous (P) and non-petroliferous (NP) areas**.

Sixty eight SDIMO alpha subunit gene sequences derived from P Soil and seventy from Np Soil were sequenced and analyzed. For a preliminary analysis, these sequences were aligned in order to detect changes in the nucleotide sequence and a phylogenetic tree was constructed (data not shown). It is worth to mention that the great majority of the sequences from the soil samples under study were clustered separately from reference sequences, showing to be more closely related to each other. The cut-off value of 83% was selected to define distinct operational protein families (OPF) based on the analysis of the phylogenetic trees of total clones in combination with Mothur analysis. Seven OPFs were defined for the P Soil, whereas only two OPFs were defined for the non-petroliferous soil. Representative sequences of distinct OPFs were submitted to analysis using the BLASTx routine. Sequences considered as belonging to the same OPF matched the same sequences from the GenBank database, with slight differences in identity values and *e*-values. It was interesting to notice that, in spite of housing such catabolic genes, the Np soil sample presented lower OPF diversity in comparison to P soil (Figure 5).

Most of the SDIMO sequences were distributed among OPFs 1 and 2 and were related to yet uncultured bacteria, being OPF 2 the most frequent one which comprised 48 sequences in P Soil and 56 in Np Soil (Table 2). All the OPFs matched SDIMO gene sequences with high similarity values (66% to 93.4%), especially with gene sequences from uncultured bacteria. Lower identity values were observed relating the query sequences to propane monooxygenase gene from the genus *Mycobacterium*. Some clones from P Soil sample contained genes identified as coding for methane monooxygenase (MmoX) from *Nocardioides *sp. and ethene monooxygenase (EtnC) from *Mycobacterium chubuense.*

**Table 2:** Blastx analyses of *SDIMO *clone sequences representing each of the OPF determined in the present study and the highest identity matches from the Genbank database.

OPF	Number of clone sequences	Gene	Best match	Average of Identity (nt)
1	5 (P) - 14 (Np)	SDIMO	Uncultured bacterium ABB70434	81.4%
2	48 (P) - 56 (Np)	SDIMO	Uncultured bacterium ABB70441	93.2%
3	3 (P)	SDIMO	Uncultured bacterium ABB70469	93.4%
4	3 (P)	EtnC	*Mycobacterium chubuense *NBB4	83.7%
5	1 (P)	SDIMO	*Mycobacterium *sp. JS623	66.0%
6	6 (P)	SDIMO	Uncultured bacterium ABB70430	77.4%
7	2 (P)	MmoX	*Nocardioides *sp. JS614	88.5%

MmoX represents Methane Monoxygenase, EtnC represents Ethene Monoxygenase, and *SDIMOs *represents soluble di-iron monoxygenase enzymes that have not been assigned to a known subgroup. Identity average shows the percentage of identity at the nucleotide level (nt) compared with sequences from Genbank database. (P) Represents sequences from P soil and (Np) from Np soil.

**Figure 6:**
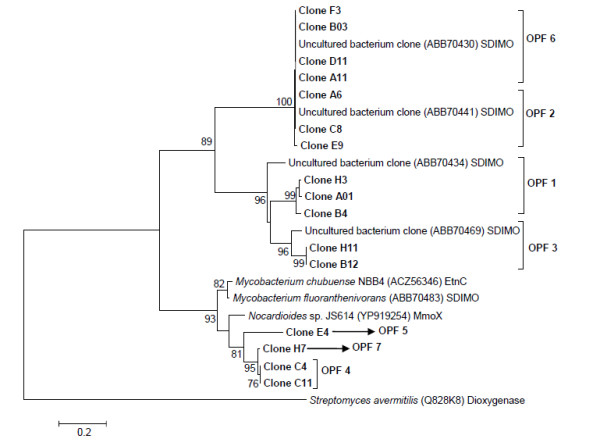
**Phylogenetic tree based on the alignment of amino acid sequences of the alpha subunit of soluble di-iron monooxygenases from clones from P soil sample and related sequences from the database**. Access numbers of *SDIMO *genes from reference strains are in brackets.

Catabolic gene sequences were translated *in silico *and representative sequences of each defined OPF were selected and together with SDIMO alpha subunit amino acid sequences from reference microorganisms were used for the tree reconstruction. Some of these sequences showed high sequence similarity levels with sequences from yet uncultured microorganisms available in the database and were recovered in tight clusters supported by high bootstrap values (Figure 6). Actually, only a few SDIMO sequences grouped with sequences from isolated microorganisms, distributed among OPFs 4, 5 and 7. These sequences showed lower similarity values, being only distantly related to known taxa such as *Nocardioides *and *Mycobacterium*. Since the reconstruction of the phylogenetic tree was based on amino acid sequences, sequences from OPFs 2 and 6, which had presented minor differences in nucleotide bases in the ClustalX alignment, were recovered in a single cluster supported by 100% bootstrap value (Figure 6). It is known that subtle changes among nucleotide sequences coding for SDIMO enzymes are considered essential because they can generate distinct biochemical functions [Cooley et al. 2009]. Therefore, based on the analyses performed using ClustalX and Mothur, the authors chose not to neglect these differences when defining the OPFs.

### Richness and Diversity Analyses

**Figure 7:**
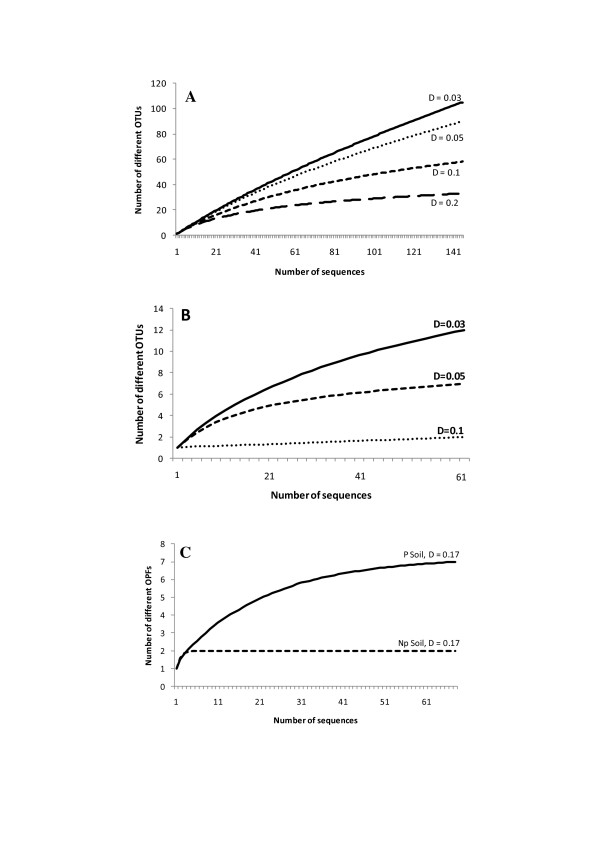
**Rarefaction analyses of 16S rRNA gene sequences from bacteria (A) and archaea (B), and of SDIMO gene sequences (C) obtained from P and Np Soil samples**.

DOTUR analyses of the 16S rDNA bacterial sequences from P soil sample allowed the definition of 105 OTUs from 145 good quality sequences, generating 83 singletons and 14 doubletons. The highest frequency OTU grouped only six sequences. Rarefaction analysis yielded an asymptotic curve at the distance level of 0.2, meaning that if more sequences were analyzed, new phyla would not have been detected (Figure 7A). However, when the distance level of species is considered (D = 0.03), it becomes clear that an increase of the sampling effort would allow the identification of new species in the data set (Figure 7A).

Diversity index and richness estimator values generated by DOTUR corroborated the great bacterial species diversity and richness that would be expected in a soil community (Table 3). In spite of the Actinobacteria abundance, the high value of Shannon index at the distance level 0.03 indicates that a great OTU diversity is also present within this phylum.

**Table 3:** Richness and diversity estimates for bacterial and archaeal 16S rRNA and SDIMO gene clone libraries from both P and Np Soil samples (sequence classification based on the cutoff value determined by DOTUR).

Gene (n^a^)	Richness^b^	Distance^c^	Chao^d^	Ace^e^	Shannon^f^
Bacterial 16S rDNA library (145)	105	0.03	331.8	391.4	4.49
Archaeal 16S rDNA library (53)	12	0.03	17	18.3	2.05
SDIMO Gene (68 - P Soil)	7	0.17	7	7.3	1.09
SDIMO Gene (70 - Np Soil)	2	0.17	2	0	0.44

^a^n, Number of gene sequences analyzed.

^b ^97% identity was estimated as the species-level distance (D = 0.03) for Bacteria and Archaea, and 83% identity for SDIMO genes.

^c^Richness is based on observed unique OTUs.

^d^Nonparametric statistical prediction of total richness of different OTUs and OPFs based on distribution of abundant (10) and rare (10) OTUs.

^e^Nonparametric statistical predictions of total richness of OTUs and OPFs based on distribution of singletons and doubletons.

^f^Shannon diversity index. A higher number represents more diversity.

The analysis of the archaeal 16S rRNA gene library, allowed the definition of 12 OTUs out of 53 good quality sequences. Rarefaction curves at the estimated phylum and order levels also reached clear saturation, suggesting that the sampling effort was sufficient to reveal all archaeal phyla and orders present in this sample (Figure 7B). Five singletons were generated in the analysis and the most frequent OTU presented 16 sequences. These results were corroborated by the Shannon diversity index and ACE and Chao richness estimator values, which were significantly lower than those found for the bacterial community (Table 3).

A total of 68 good quality sequences derived from the catabolic gene libraries from P Soil were analyzed and seven distinct protein groups were obtained based on the distance level previously defined for OPFs, which were later confirmed by Blast score ratio analysis performed in BlastP database. For the Np soil sample, 70 sequences were analyzed and only two protein groups could be defined and confirmed. Rarefaction analysis performed for the SDIMO gene library from both samples yielded plateau-shaped curves, indicating that the saturation of the gene diversity in the environment was complete and the sampling effort was satisfactory, in particular for the Np soil sample (Figure 7C).

### Real time quantitative PCR

**Figure 8:**
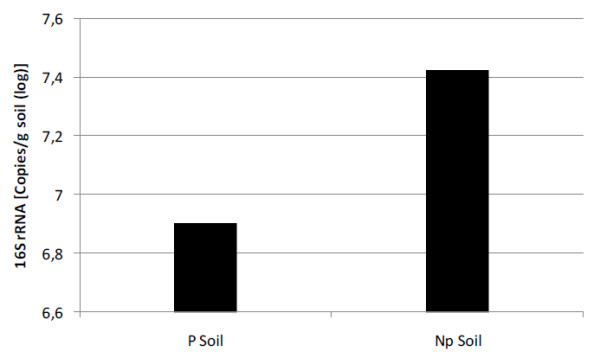
**Quantification of bacterial 16S rRNA gene in both soil samples analyzed**. P soil indicates the soil at Potiguar Basin, and Np soil indicates the non-petroliferous soil.

With the use of the real-time PCR technique it was possible to successfully construct the bacterial 16S rRNA gene standard curve, achieving satisfactory indices for bacterial quantification (R2 = 0.99, efficiency = 94%). The DNA sample representing the target to be quantified reached the interval of the generated curve among the points from the dilution series. The log copy number calculated was 6.901/g and 7.427/g soil for P Soil and Np Soil, respectively (Figure 8).

The application of this technique aimed at the quantification of the total bacterial population, through the 16S rRNA gene copy number, and the quantification of specialized populations of microorganisms that consume short-chain hydrocarbons, through the SDIMO gene quantification. Such analysis would have generated the proportion of potentially degrading bacterial population in relation to the total community. However, quantification of SDIMO genes yielded a very low copy number, which was probably under the detection level of the analytic procedure.

## Discussion

Prokaryotic community diversity and abundance were investigated in a soil sample originated from a sedimentary basin by means of 16S rRNA and catabolic gene libraries in combination with real-time PCR. Results revealed the outstanding high abundance of the phylum Actinobacteria in the bacterial community, especially the order Actinomycetales, an important group inhabiting the soil environment that encompasses the genera from the CRNM complex, known for its hydrocarbon degradation skills [Shennan 2006].

In general, most of the groups identified in the samples are of common occurrence in the environment, especially in soils and sediments. In the Actinobacteria phylum, known for its ability of surviving under adverse conditions, the genus *Saccharopolyspora *had a considerable contribution. Clones were affiliated to the species *Saccharopolyspora rosea*, which was first isolated from a patient with bronchial carcinoma [Yassin 2009], and other species that generally occur in the environment, such as saline lakes, soil, marine sediments and even sponge tissue: *Saccharopolyspora gloriosa, Saccharopolyspora cebuensis, Saccharopolyspora antimicrobica, Saccharopolyspora salina *and *Saccharopolyspora halophila *[Pimentel-Elardo et al. 2008; Yuan et al. 2008; Suthindhiran & Kannabiran 2009; Tang et al. 2009; Qin et al. 2010]. Some other species belonging to the same order, Actinomycetales, included *Actinopolyspora xinjiangensis*, an extreme halophile isolated from salt-lake sediments [Guan et al. 2010], *Nocardioides aestuarii*, isolated from tidal flat sediment [Yi and Chun 2004] and *Jonesia denitrificans*, a pathogenic organism that had its genome sequencing completed in 2009 [Pukall et al. 2009].

Some genera and species more specifically related to the metabolism of hydrocarbons were also recovered, such as *Mycobacterium pallens*, a polycyclic aromatic hydrocarbon-degrader isolated from Hawaiian soils [Hennessee et al. 2009], *Mycobacterium *sp. TY-6, known for its capacity of degrading propane [Kotani et al. 2006] and *Nocardioides *sp., an alkene-degrading bacteria [Vomberg and Klinner 2000]. In this sense, there was also a contribution of organisms from the phylum Proteobacteria, such as uncultured bacteria from the family Burkholderiaceae (Betaproteobacteria), which comprises oil degrading lineages and cultures reported to degrade both aliphatic and aromatic hydrocarbons [Chakraborty et al. 2010], and an *Enterobacter *sp. strain (Gammaproteobacteria), which is known for its ability of degrading methyl tert-butyl ether (MTBE), a gasoline constituent [Kao et al. 2010]. The order Desulfuromonadales (Deltaproteobacteria), constituted mainly by anaerobic bacteria [Thomas et al. 2008], was also detected. This group encompasses mainly sulfate-reducing bacteria [Borin et al. 2009] and several species possessing this metabolism may present in addition the ability of degrading short-chain hydrocarbons [Kniemeyer et al. 2007].

The group *Vibrio *was present in the sample and represented mainly by the species *Vibrio chagasii *and *Vibrio splendidus*. Organisms belonging to this genus often move from mutualistic to pathogenic associations with aquatic animals [Lacoste et al. 2001; Gomez-Leon et al. 2005; Venier et al. 2011]. In a recent study, Chien and co-workers (2007) identified *Vibrio *strains from mangrove sediment able to accumulate polyhydroxyalkanoates (PHAs). These compounds are polyesters accumulated by various bacteria under unbalanced growth conditions which can be useful for the development of non-petroleum based biodegradable plastics [Chien et al. 2007].

Clones related to the genus *Paenibacillus *(phylum Firmicutes) were also found in the 16S rDNA gene library from P soil sample. Bacteria from this phylum are ubiquitous and have the ability to adapt to different types of environment and generally present high abundance in samples from anaerobic digesters, anaerobic sludge, landfill and fecal samples [Krause et al. 2008; Krober et al. 2009], suggesting the ability to consume complex organic compounds, such as polysaccharides, being essential for organic matter breakdown process and methane production.

Representants of Acidobacteria were identified in P soil sample only as uncultured organisms. The majority of the bacteria from this group are uncultured and only known by their 16S rDNA sequences [Quaiser et al. 2003]. Based on their phylogenetic diversity and ecological distribution, Acidobacteria are expected to represent a metabolically and genetically diverse group comparable to other well known phyla and are currently found in soils, freshwater habitats, hot springs, wastewaters and others [Quaiser et al. 2003].

Gemmatimonadetes-related clones identified in the P soil library presented high sequence similarity to an organism isolated from a community of a semiarid lead-zinc mine tailing site [Mendez et al. 2008]. Representatives of this group are generally isolated from alkaline environments with moderate temperatures and aerobic conditions, and also present low substrate versatility [Hartman 2009].

In spite of the great bacterial diversity frequently found in soils, and also supported in the present work, it is possible to recognize a certain pattern regarding the contribution of each phylum, being the great majority of clones usually affiliated to nine major bacterial phyla and generating, to a certain extent, stability in the community structure. According to Janssen (2006), dominant phyla usually correspond to approximately 92% of the libraries, being Proteobacteria and Acidobacteria the most abundant ones, while Actinobacteria would only represent about 13% of total clones. Phylogenetic analysis of bacterial community from Potiguar petroliferous basin soil sample showed a different distribution in comparison to the general pattern. Actinobacteria had a contribution of more than half of the library, while Proteobacteria and Acidobacteria represented only 12% and 11%, respectively. Several theories have been suggested to explain the significant occurrence of Actinobacteria in environmental samples, being resource limitation the most accepted one, such as the ability to adapt to recalcitrant substrate [Fierer et al. 2007].

The calculation of the Shannon index reinforced the observation that the P soil sample harbors a highly diverse bacterial community. Shannon values found in the literature for bacterial communities ranged from 2.96 to 3.83 in libraries from water column [Dimitriu et al. 2008]; 2.7 to 3.5 in coral samples [Bourne et al. 2008], 3.6 to 4.0 for refinery WWTP sludge [Silva et al. 2010] and a diversity index of 2.9 was obtained for a petroliferous reservoir sample [Li et al. 2007]. According to ecological theories more diverse communities may offer a more significant contribution to the ecosystem functions, keeping in mind that ecological studies on soil microbial communities have revealed that this type of habitat generally presents extreme diversity, with species richness estimates ranging from values of 2000 to 52000 [Youssef & Elshahed. 2009].

A great number of clones from both libraries showed high similarity levels to gene sequences originated from uncultured bacteria and archaea of different environmental samples, such as deep-lake sediment, springs, tropical forest plants, pasture, agricultural soil, marine biofilm, rhizosphere, urban aerosol and bioreactors, suggesting the cosmopolitan distribution of these organisms in the environment.

Phylogenetic analysis of archaeal 16S rRNA gene sequences showed a high abundance of organisms identified as *Candidatus nitrososphaera gargensis*. This group was considered the first member of Crenarchaeota I.1b group in which ammonium oxidation could be observed at the cellular level [Hatzenpichler et al. 2007]. Due to their phylogenetic and metabolic characteristics these organisms are now classified as members of the phylum Thaumarchaeota, which encompasses mesophilic archaea previously affiliated to Crenarchaeota and with a major role in geochemical cycles [Brochier-Armanet et al. 2008; Spang et al. 2010]. *Nitrososphaera gargensis *is a representative of an evolutionary lineage within the group Thaumarchaeota that occurs predominantly in terrestrial environments.

Nonetheless, sequence identification through sequence databases offered significant limitations in the present study and identification at species level turned out to be a very difficult task for a large quantity of sequences. Analysis based on 16S rRNA gene sequencing can be very useful in surveying dominant groups in samples under study, however it can be inefficient in detecting and identifying species related to light gas metabolism, already known for its low abundance in natural environments [Coleman et al. 2006]. Therefore, the analysis of microbial community composition based on 16S rRNA genes libraries of relative small size, in spite of representing a fundamental method for a general characterization of microbial communities, seems to be inefficient for a more specific application such as the microbial prospection of oil and gas. The detection of organisms that correspond to the most abundant OTUs requires minimal sampling, whereas the recovery of sequences from minor components demands surveys that are many orders of magnitude larger than those reported in the literature. These low-abundance organisms that represent rare OTUs constitute the so called "rare biosphere", which is still largely unexplored. According to Sogin (2006), the rare biosphere may serve as a potentially inexhaustible reservoir of genomic innovation and its members usually have a sparse distribution in nature. Recent high-sensitivity surveys of diverse environmental samples have revealed that the vast majority of the microbial diversity in a specific habitat is comprised by taxa that are present at very low abundances and the most abundant organisms represent only a fraction of the total diversity [Brazelton 2010]. In a recent work, Galand and co-workers (2009) demonstrated patterns of biogeography for the rare biosphere and concluded that its diversity is most likely subjected to ecological processes such as selection, speciation, and extinction.

In this context, the use of 16S rRNA gene libraries for the investigation of microbial populations responsible for short-chain alkane degradation, mainly ethane and propane, may be time consuming and offer low resolution in terms of species and genes as key players in the degradation processes. The analysis of catabolic gene libraries may offer a more appropriate technique to be applied to MPOG, considering that it is directly focused on light gas metabolism. The present study revealed that most of the SDIMO genes recovered from soil samples correspond to potentially new genes, which are not related to those found in known bacterial species, a fact that made it difficult to infer which microbial group carried the short-chain alkane degradation genes. SDIMOs subgroups 3, 4, 5 and 6 could be identified in the soil samples under study. The six known subgroups are phylogenetically distinct and present significant evolutionary distance among each other. These large evolutionary gaps suggest that known SDIMOs are not representative of the naturally occurring diversity, what makes the group a worthy target for bioprospecting [Holmes & Coleman 2008]. According to OPFs definition and Mothur analysis it could be inferred that there are fewer lineages of microorganisms involved directly in the metabolism of short-chain hydrocarbons in a non-petroliferous soil.

Hydrocarbon levels measured in P and Np soil samples through gas chromatography did not detect a geochemical anomaly on soil from petroliferous basin as would be expected. Some characteristics from soils have been proved to affect this type of analysis. According to Horvitz (1985) the acidic nature of some soils may prevent the hydrocarbons from being adsorbed, therefore making the real levels that occur in nature different from those measured in laboratory. Another problem is the presence of large quantities of biogenic methane in most near-surface soils, which can confuse the interpretation of gas levels related to the leakage from buried reservoirs.

One of the first works on MPOG based on molecular biological methods, carried out by Zhang and co-workers (2010), focused on soil methanotrophic populations as a bioindicator for the presence of oil and gas in a field in China. In spite of the successful quantification of such organisms by real-time PCR, the common presence of biogenic methane in soil represents an obstacle to link the occurrence of methanotrophic populations to the presence of oil and gas reservoirs. Since methane is a common product of microbial action on organic matter, the presence of methanotrophic organisms in a given sample is less probable of representing an indicative of a reservoir than the occurrence of more specific bacterial populations, such as ethane, propane and butane degraders [Shennan 2006]. In addition, short-chain hydrocarbon degrading bacteria from the CNMR complex, which carry the SDIMO genes, posses several physiological features that make these organisms a more appropriate bioindicator in microbial prospection methods. Slow development in low-nutrient conditions, a peculiar cell wall highly resistant to desiccation, capacity of surviving in starving conditions for long periods and an adjustable endogenous metabolism are some skills that can explain the surviving success of this group.

In conclusion, the results gathered in this work suggest that cultivation-independent microbial molecular techniques in combination with the light hydrocarbon degrading bacterial populations as a target will improve the accuracy rate of MPOG. Further studies on the occurrence and diversity of SDIMO genes in soil, as well as the improvement of primer sets to be applied in real-time PCR, are necessary in order to overcome the obstacle of the low abundance of catabolic genes in natural environments and enable their quantification from complex genetic backgrounds.

## Competing interests

The authors declare that they have no competing interests.
